# Interface designed MoS_2_/GaAs heterostructure solar cell with sandwich stacked hexagonal boron nitride

**DOI:** 10.1038/srep15103

**Published:** 2015-10-13

**Authors:** Shisheng Lin, Xiaoqiang Li, Peng Wang, Zhijuan Xu, Shengjiao Zhang, Huikai Zhong, Zhiqian Wu, Wenli Xu, Hongsheng Chen

**Affiliations:** 1 Department of Information Science and Electronic Engineering, Zhejiang University, Hangzhou, 310027, China; 2 State Key Laboratory of Modern Optical Instrumentation, Zhejiang University, Hangzhou, 310027, China

## Abstract

MoS_2_ is a layered two-dimensional semiconductor with a direct band gap of 1.8 eV. The MoS_2_/bulk semiconductor system offers a new platform for solar cell device design. Different from the conventional bulk p-n junctions, in the MoS_2_/bulk semiconductor heterostructure, static charge transfer shifts the Fermi level of MoS_2_ toward that of bulk semiconductor, lowering the barrier height of the formed junction. Herein, we introduce hexagonal boron nitride (h-BN) into MoS_2_/GaAs heterostructure to suppress the static charge transfer, and the obtained MoS_2_/h-BN/GaAs solar cell exhibits an improved power conversion efficiency of 5.42%. More importantly, the sandwiched h-BN makes the Fermi level tuning of MoS_2_ more effective. By employing chemical doping and electrical gating into the solar cell device, PCE of 9.03% is achieved, which is the highest among all the reported monolayer transition metal dichalcogenide based solar cells.

Two-dimensional (2D) materials provide rich physics in designing of new optoelectronic devices^1^,^2^,^3^,^4^. Because of the low light absorbance of the atomic thin 2D materials^5^,^6^, the external semiconductor is usually incorporated to improve the performance of 2D material based devices^7^,^8^,^9^. Photodetectors based on monolayer graphene have been reported to show photo gain as high as ~10^8^ and photo responsivity as high as ~10^7^ A/W through the enhanced light absorption with covering semiconductor quantum dots on graphene^8^. Forming 2D materials/bulk materials heterostructure junctions is an alternative choice to obtain high performance optoelectronic devices as the bulk semiconductor can fully absorb incident light^10^,^11^. As the first discovered 2D material with many fascinating electrical and optical properties, graphene and its heterostructures have attracted much attention for solar cells worldwide^12^,^13^,^14^,^15^. Power conversion efficiency (PCE) of solar cells based on graphene/Si system has been improved from 1.65% to 15.6% since the first reported graphene/Si heterostructure solar cell in the year 2010^16^,^17^. Recently, we have reported graphene/GaAs solar cell with PCE of 18.5%^18^. On the other hand, single layer 2D molybdenum disulfide (MoS_2_) is semiconductor with a direct band gap of 1.8 eV^19^. MoS_2_ with thickness less than 1 nm can absorb 5–10% incident light^20^. Also, MoS_2_ can be synthesized with large area by chemical vapor deposition (CVD) method^21^,^22^,^23^. Based on the abovementioned merits, the MoS_2_/bulk semiconductor system offers a new platform for optoelectronic device design. It has been reported that MoS_2_/Si heterostructure solar cell has an efficiency of 5.23% with the assistance of aluminum deposition on MoS_2_^6^. However, much more work on MoS_2_/semiconductor heterostructure is highly desirable both for the fundamental research interest and the potential photovoltaic application. Among all the bulk semiconductors, GaAs has a suitable direct band gap of 1.42 eV and high electron mobility (8000 cm^2^V^−1^s^−1^ at 300 K)^24^, which makes itself one of the best candidates for high performance solar cells^25^,^26^.

Tunable Fermi level is one of the unique physical properties of 2D materials, which can be finely tuned by chemical doping or electrical gating^27^,^28^,^29^,^30^. Different from the conventional bulk p-n junctions, there is static charge transfer between 2D materials and bulk semiconductor, which could severely lower the Fermi level difference between bulk semiconductor and 2D material^31^, and lead to a decreased junction barrier height. The photovoltaic performance of the heterojunction is greatly influenced by the junction barrier height, which means suppressing the static charge transfer between 2D materials and semiconductor substrate are highly desirable. Herein, we introduce 2D hexagonal boron nitride (h-BN) into the MoS_2_/GaAs heterostructure to suppress the static charge transfer. More importantly, the inserted h-BN layer makes the tuning of Fermi level of MoS_2_ more effective, which greatly improves the performance of solar cells. Based on the interface band structure designing and Fermi level tuning of MoS_2_, 9.03% of PCE has been achieved.

## Results

### Physical design of the MoS_2_ based solar cell

**Figure 1:**
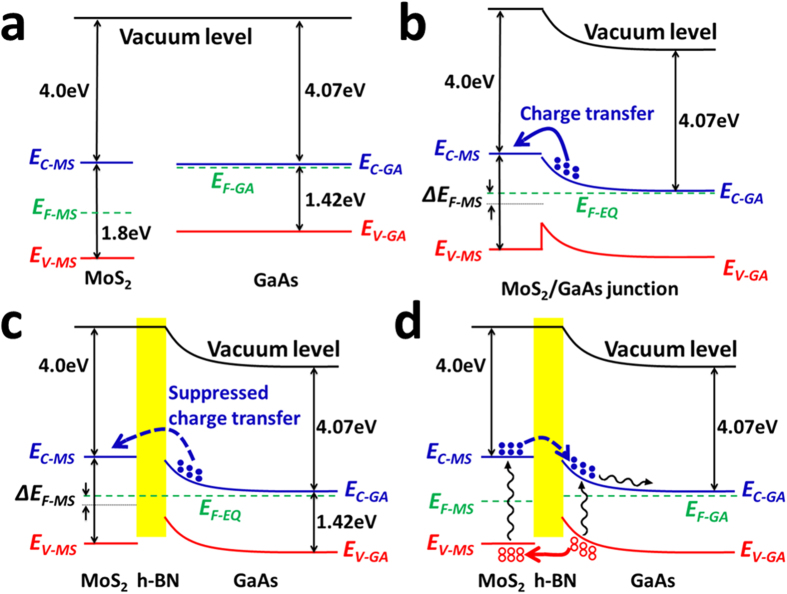
Schematic electronic band structure of independent MoS_2_ and GaAs (a), MoS_2_/GaAs Schottky junction (b), MoS_2_/h-BN/GaAs heterojunction (c) under equilibrium condition and the electronic band structure of MoS_2_/h-BN/GaAs heterojunction under illumination (d).

The schematic electronic band structure of the independent MoS_2_ and GaAs is shown in Fig. 1a. The electron affinity (energy gap between vacuum level and the bottom level of conduction band E_C-MS_) of MoS_2_ (*χ*_MS_) is 4.0 eV^32^, and the band gap of MoS_2_ is 1.8 eV. As the measured sheet resistance of MoS_2_ is in the range of 10^4^–10^6^ Ω/□, indicating the Fermi level of MoS_2_ (E_F-MS_) locates near the middle of the band gap. The electron affinity of GaAs (*χ*_GA_) is 4.07 eV. The Fermi level of GaAs (E_F-GA_) used in this study locates around the bottom level of conduction band (E_C-GA_) because the n-type doping concentration is around 10^18^ cm^−3^. When MoS_2_ touches with GaAs, due to the Fermi level difference, some of majority electrons of GaAs inject into MoS_2_, shifting E_F-MS_ by ΔE_F-MS_, as shown in Fig. 1b, which can be quantitatively expressed as:


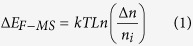


where Δn is the change of electron concentration in MoS_2_ which affected by the injected electrons from GaAs, *n*_*i*_ is the intrinsic carrier concentration in MoS_2_, *k* is the Boltzmann constant and *T* is the absolute temperature. The barrier height (*Φ*_*barrier*_) of the MoS_2_/GaAs heterojunction can be presented as:


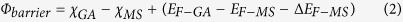


It is very clear that suppressing the static charge transfer during the formation of MoS_2_/GaAs heterojunction can result in higher *Φ*_*barrier*_. We propose a device by inserting 2D h-BN into MoS_2_/GaAs Schottky diode as the interface layer to suppress the static charge transfer. h-BN is one of 2D materials with a band gap of 5.9 eV and dielectric constant of 4.0^33^. The electronic band alignment of MoS_2_/h-BN/GaAs heterojunction can be seen in Fig. 1c. As h-BN has a negative electron affinity^34^, the electron transfer from GaAs to MoS_2_ is suppressed during the formation of the MoS_2_/h-BN/GaAs heterojunction. As a result, ΔE_F-MS_ is reduced and *Φ*_*barrier*_ of the junction is lifted up. Under illumination, photo generated excess electrons and holes are collected by GaAs and MoS_2_, respectively. As shown in Fig. 1d, transport of holes from GaAs to MoS_2_ is almost unaffected after inserting the ultrathin 2D BN layer, which dominates the power conversion from light to electricity. In other words, the open circuit voltage (*V*_*oc*_) of the solar cell can be increased by the inserted h-BN while short circuit current density (*J*_*sc*_) stays almost unchanged, thus, solar cell with a better performance can be expected.

**Figure 2:**
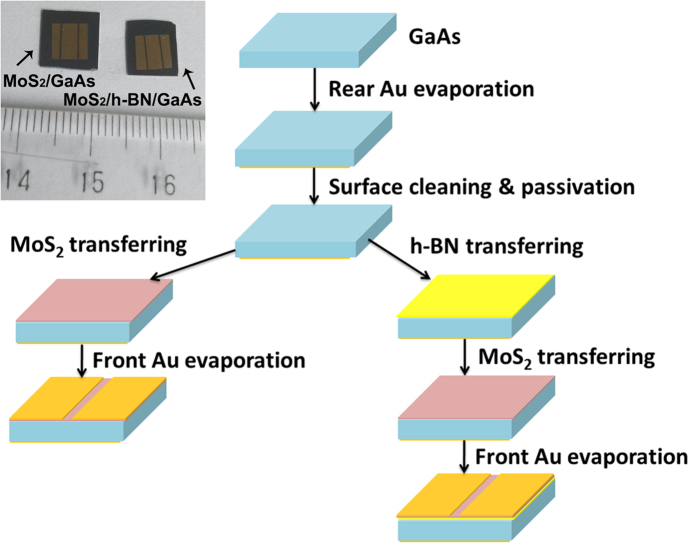
Schematic fabrication processes of MoS_2_/GaAs and MoS_2_/h-BN/GaAs Schottky junction based solar cells. The up-left inset shows the digital photographs of the corresponding devices. shows the digital photographs of the corresponding devices.

Fig. 2 shows the schematic fabrication processes of MoS_2_/GaAs and MoS_2_/h-BN/GaAs Schottky junction based solar cells. After removal of the native oxide on the GaAs substrate, Au with a thickness of 60 nm was evaporated on the rear surface of GaAs forming ohmic contact. Then the front surface of GaAs was cleaned with dilute HCl aqueous solution. Front surface passivation is achieved with remote NH_3_ plasma treatment for 5 min with power of 120 Watt and frequency of 27.5 MHz. After the passivation treatment, h-BN and MoS_2_ in sequence or MoS_2_ alone is directly transferred onto the front surface of GaAs substrate, followed with the deposition of front Au contacts (60 nm) with mask. Inset in Fig. 2 shows the digital photographs of the typical MoS_2_/GaAs and MoS_2_/h-BN/GaAs heterojunction based solar cells, where the thin line shape of the active area can be seen, which is designed for efficiently current collection based on the high resistance of monolayer MoS_2_.

### Basic properties of the MoS_2_/h-BN/GaAs heterostructure solar cell

**Figure 3:**
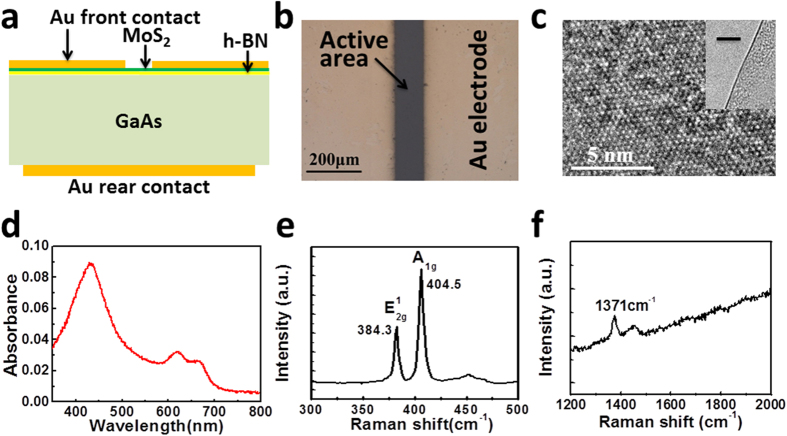
(**a**) Schematic structure of the MoS_2_/h-BN/GaAs heterostructure. (**b**) Optical micrograph of the MoS_2_/h-BN/GaAs solar cell. (**c**) HRTEM of the monolayer MoS2, inset is the TEM image of the edge of the MoS_2_ layer, where the bar represents 10 nm. (**d**) Absorption spectrum of the monolayer MoS_2_. Raman spectra of monolayer MoS_2_ (**e**) and monolayer h-BN (**f**) used in this study.

Fig. 3a shows the schematic cross section structure of the MoS_2_/h-BN/GaAs heterojunction solar cell, which is composed of rear Au contact, GaAs substrate, h-BN layer, MoS_2_ layer and the front Au contact from bottom to top. The active area of the device is defined with the opened window in the front Au contact, as shown in Fig. 3b. The width of the active area is 120 μm and the length is 5 mm, making the active area 0.6 mm^2^. As the thickness of the front Au contact is 60 nm, no light can be absorbed by the device in the Au shadowed area, which guarantees the precise active area. The high resolution transmission electron microscopy (HRTEM) image of the MoS_2_ is shown in Fig. 3c, which shows the six fold symmetry nature of the MoS_2_. The inset of the Fig. 3c shows the HRTEM image of MoS_2_ layer, which clearly indicates the CVD grown MoS_2_ is monolayer. The electron diffraction pattern can be seen in Supplementary Information Fig. S1, which also implies the monolayer nature of MoS_2_. Fig. 3d shows the absorption spectrum of the CVD grown MoS_2_, where three absorption peaks corresponding to 436 nm, 619 nm and 662 nm can be seen in the wavelength range of 350–800 nm. The peak of the absorbance locates at 436 nm is 8.9%, which is in agreement with the reported absorbance of the monolayer MoS_2_^20^. The Raman spectrum of the MoS_2_ on Si/SiO_2_ substrate is shown in Fig. 3e. The Raman peaks corresponding to E^1^_2g_ and A_1g_ modes of MoS_2_ locate at 384.3 cm^−1^ and 404.5 cm^−1^, respectively, indicating the grown MoS_2_ is monolayer^35^. Fig. 3f presents the Raman spectrum of h-BN, where the 1371 cm^−1^ peak indicates it is monolayer^36^. The digital photographs of transferred h-BN on Si/SiO_2_ substrate, optical microscopy image and atomic force microscopy image of MoS_2_ and optical microscopy image of h-BN can be seen in Supplementary Information Fig. S2, where can be seen that the homogeneity of CVD grown MoS_2_ and h-BN is good.

**Figure 4:**
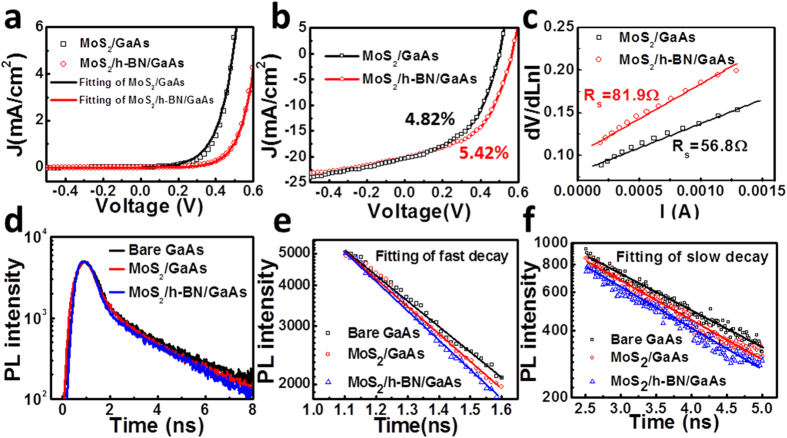
(**a**) Dark J-V curves of the MoS_2_/GaAs and MoS_2_/h-BN/GaAs heterojunctions. (**b**) J-V curves of the MoS_2_/GaAs and MoS_2_/h-BN/GaAs heterojunctions under AM1.5G illumination. (**c**) Linear fitting of dV/dLnI~I data for obtaining R_s_ of the devices. (**d**) Transient PL of the bare GaAs substrate, MoS_2_/GaAs and MoS_2_/h-BN/GaAs heterojunctions. (**e**) Fitting of the PL decay time constant in the fast decay range and (**f**) in the slow decay range.

Fig. 4a shows the dark current density-voltage (*J*-*V*) curves of the MoS_2_/GaAs and MoS_2_/h-BN/GaAs heterojunctions, both of which show good rectifying characteristics. It is noteworthy that in this study if no mentioned, GaAs substrate is n-type doped. We also test the *J*-*V* curve of MoS_2_/p-GaAs, which shows bad rectifying characteristics as presented in Supplementary Information Figure S3. The threshold voltage (the voltage needed to reach a current density of 2 mA/cm^2^ here) for the MoS_2_/GaAs heterojunction is 0.41 V, while the value for the MoS_2_/h-BN/GaAs hetrostructure is 0.52 V, suggesting that *Φ*_*barrier*_ is increased by the interlayer h-BN. The value of *Φ*_*barrier*_ can be deduced through fitting of dark *J*-*V* curves as expressed by:


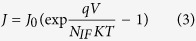


where *K* is the Boltzmann constant, *N*_*IF*_ is the junction ideality factor and *q* is the value of electron charge. Based on thermionic-emission theory, saturation current density *J*_*0*_ can be described as:


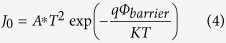


where A^*^ is the effective Richardson’s constant of n-type GaAs (8.16 A/k•cm^2^)^37^. Based on equations (3) and (4), the values of *N*_*IF*_ for MoS_2_/GaAs and MoS_2_/h-BN/GaAs heterojunctions are 3.18 and 2.73, and the *Φ*_*barrier*_ values are 0.71 eV and 0.78 eV, respectively. The lower *N*_*IF*_ of the MoS_2_/h-BN/GaAs device compared with that of the MoS_2_/GaAs device indicates that the interface recombination rate is decreased with interlayer h-BN. Fig. 4b shows the *J*-*V* curves of the MoS_2_/GaAs and MoS_2_/h-BN/GaAs heterostructure solar cell under AM1.5G illumination. As *Φ*_*barrier*_ is increased, with interlayer h-BN, *V*_*oc*_ of the solar cell is increased from 0.51 V to 0.57 V, while the *J*_*sc*_ is slightly decreased from 20.6 mA/cm^2^ to 20.2 mA/cm^2^. With the values of fill factor (FF, FF = P_max_/(V_oc_ × J_sc_)) as 45.9% and 47.0%, the PCE values (PCE = V_oc_ × J_sc_ × FF) for the MoS_2_/GaAs and MoS_2_/h-BN/GaAs solar cells are 4.82% and 5.42%, respectively. The series resistance (*R*_*s*_) fitting results of the MoS_2_/GaAs and MoS_2_/h-BN/GaAs solar cells are shown in Fig. 4c, which demonstrates *R*_*s*_ is increased from 56.8 Ω for the MoS_2_/GaAs device to the value of 81.9 Ω for the MoS_2_/h-BN/GaAs device. FF is mainly influenced by *N*_*IF*_ and *R*_*s*_. Lower *N*_*IF*_ leads to higher FF while higher *R*_*s*_ leads to lower FF. For the MoS_2_/h-BN/GaAs device, even with increased *R*_*s*_, the decreased *N*_*IF*_ increases the FF compared with the value of MoS_2_/GaAs device.

The electrical properties mentioned above indicate the importance of the interface recombination condition for the MoS_2_/GaAs and MoS_2_/h-BN/GaAs devices. Here transient photoluminescence (PL) is employed to investigate the kinetics of the photo generated carriers near the interface. Fig. 4d shows the transient PL decay curves for bare GaAs substrate, and the same MoS_2_/GaAs and MoS_2_/h-BN/GaAs heterostructure devices with PCE of 4.82% and 5.42% respectively. The decay curves show double channel dependent behavior, corresponding to a fast decay channel (in the range of 1 ns to 2 ns) and a slow decay channel (after 2 ns). The wavelength of the excitation laser is 450 nm, and the absorption depth is close to the surface of GaAs (about 50 nm). The quick decay range in the first nanosecond is related to carrier kinetics at surface or interface, and the subsequent slow decay range is dominated by the bulk recombination processes. PL decay time constants are deduced by exponentially fitting the PL intensity decay curves in the fast and slow decay ranges as shown in Fig. 4e,f, respectively. The fitted PL decay time constants in the fast decay range for bare GaAs, MoS_2_/GaAs and MoS_2_/h-BN/GaAs are 0.97 ns, 0.59 ns and 0.52 ns, respectively, and the values in the slow decay range are 1.92 ns, 1.89 ns and 1.85 ns, respectively. For the bare GaAs, photo generated excess carriers recombine with emission of photons or phonons. In MoS_2_/GaAs heterostructure, besides the process mentioned above, parts of the excited holes in GaAs are separated by the heterojunction and collected by MoS_2_. The separated electrons and holes cannot participate in the radiation recombination process. Thus PL decay time constant is decreased. In the MoS_2_/h-BN/GaAs heterostructure, *Φ*_*barrier*_ is increased, which leads to higher speed of carrier separation process and even shorter PL decay time constant. Recombination will take place when electrons produced in GaAs cross the interface. The recombination rate is influenced by the carriers crossing time, which corresponds to the PL decay time constant in the fast decay range. For the device with interlayer h-BN, crossing time is shortened, resulting in the lowered interface recombination rate and lower value of *N*_*IF*_. For the PL decay in the slow decay range, similar time constants imply that the PL decay process is dominated by the bulk recombination properties.

### Enhance the performance of the MoS_2_/h-BN/GaAs heterostructure solar cell by chemical doping

**Figure 5:**
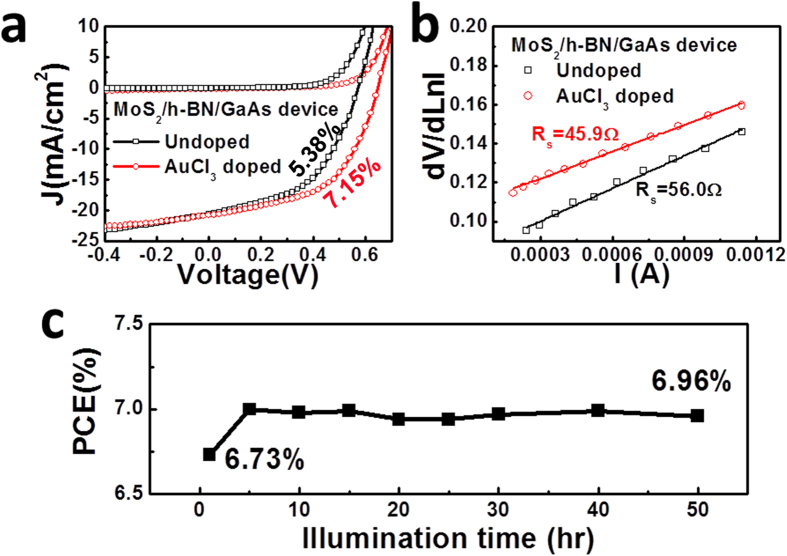
(**a**) *J*-*V* curves in the dark and under AM1.5G illumination of undoped and doped MoS_2_/h-BN/GaAs solar cells. (**b**) *R*_*s*_ fitting of the MoS_2_/h-BN/GaAs solar cell device with and without AuCl_3_ doping. (**c**) Performance stability of the MoS_2_/h-BN/GaAs solar cell under AM1.5G illumination.

The Fermi level and carrier concentration in MoS_2_ can be tuned by chemical doping. In this study, AuCl_3_ solution in nitromethane (1 mM) is used to doping 2D MoS_2_ to increase the PCE of the MoS_2_/h-BN/GaAs solar cell. The *J*-*V* curves in the dark and under AM1.5G illumination can be found in Fig. 5a. The threshold voltage increases from 0.45 V to 0.58 V for after doping of MoS_2_, indicating *Φ*_*barrier*_ increases by AuCl_3_ doping of MoS_2_. The value of *Φ*_*barrier*_ can be deduced through fitting of dark *J*-*V* curves based on equation (3) and (4). The obtained values are 0.79 eV and 0.85 eV for the undoped and doped MoS_2_/h-BN/GaAs devices, respectively. As *Φ*_*barrier*_ increases, *V*_*oc*_ of the MoS_2_/h-BN/GaAs heterostructure based solar cell increases from 0.56 V to 0.64 V. Meanwhile, *J*_*sc*_ is slightly increased from 20.6 mA/cm^2^ to 20.8 mA/cm^2^. The FF values are 46.6% and 53.7%, and the PCE values are 5.38% and 7.15% for the undoped and doped devices, respectively. The increase of the FF after doping is related to the decrease of *R*_*s*_, as shown in Fig. 5b, the value of *R*_*s*_ without doping of MoS_2_ is 56.0 Ω, while *R*_*s*_ is decreased to 45.9 Ω after doping. In addition, to explore the stability of the doped MoS_2_/h-BN/GaAs solar cell, we test the variation of the PCE values in 50 hours under AM1.5G illumination, as seen in Fig. 5c. The device was sealed by polymethyl methacrylate (PMMA) through spining-on-coating. The starting PCE value is 6.73%, while after illumination for 50 hrs, the PCE increases to 6.96%. Considering the light induced degradation of crystaslline silicon solar cell is usually happened in the first 24 hrs under AM1.5G illumination, it can be safely concluded the stability of MoS_2_/h-BN/GaAs solar cell under illumination is good with suitable encapsulation.

### Further improvement of the MoS_2_/h-BN/GaAs heterostructure solar cell by electrical gating

**Figure 6:**
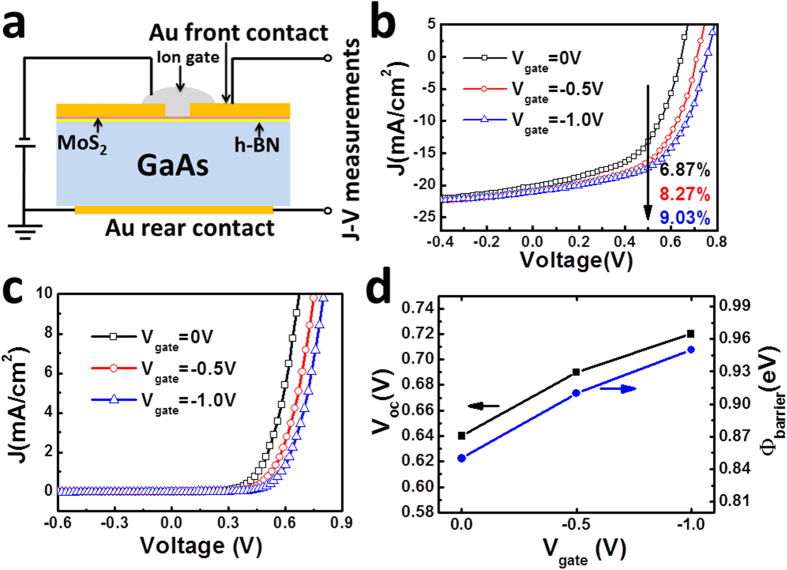
(**a**) Schematic structure of the field effect MoS_2_/h-BN/GaAs solar cell. (**b**) *J*-*V* curves of the field effect MoS_2_/h-BN/GaAs heterojunctions under AM1.5G illumination and different *V*_*gate*_. (**c**) Dark *J*-*V* curves of the field effect MoS_2_/h-BN/GaAs heterojunctions under different *V*_*gate*_. (**d**) Values of *Φ*_*barrier*_ and *V*_*oc*_ correspond to different *V*_*gate*_.

As a atomic thin 2D semoconductor, the Fermi level of MoS_2_ can be finely tuned with gating effect^38^. Here we employ PEO based ion polymer as the top gate electrode^39^, and the schematic structure of the field effect MoS_2_/h-BN/GaAs solar cell is shown in Fig. 6a. Ion gate is directly covered on the surface of AuCl_3_ doped MoS_2_. Negative voltage is applied on the ion gate and the rear Au contact is connected to the ground. Fig. 6b shows the *J*-*V* curves of the field effect solar cell under AM1.5G illumination. When gate voltage (*V*_*gate*_) equals to −0.5 V, *V*_*oc*_ of the solar cell is increased from 0.64 V to 0.72 V. Meanwhile, *J*_*sc*_ is slightly increased from 20.2 mA/cm^2^ to 20.7 mA/cm^2^, which might be attributed to the enhanced efficiency of charge seperation with gating. FF is increased from 53.1% to 54.9% and PCE is improved from 6.87% to 8.27%. when *V*_*gate*_ equals to −1.0 V, the obtained values of *V*_*oc*_, *J*_*sc*_ and FF are 0.76 V, 21.1 mA/cm^2^ and 56.3%, respectively. And the final PCE is 9.03%. Fig. 6c shows the dark *J*-*V* curves with different *V*_*gate*_. The threshold voltage is increased when the negative *V*_*gate*_ increases. By fitting of the dark *J*-*V* curves shown in Fig. 6c, values of *Φ*_*barrier*_ can be obtained and shown in Fig. 6d, where the values of *V*_*oc*_ corresponding to different *V*_*gate*_ are also shown. Fig. 6d discloses that the increased *V*_oc_ is mainly attributed to the improved *Φ*_*barrier*_ under gating effect. The *Φ*_*barrier*_ values are 0.85 eV, 0.91 eV and 0.95 eV corresponding to the *V*_*gate*_ values of 0 V, −0.5 V and −1.0 V, respectively. *V*_*gate*_ higher than −1.0 V causes gate leakage current in this experiment, causing the dark *J*-*V* curves away from the (0, 0) point and the *J*-*V* curves under illumination unreliable. Thus no further *J*-*V* curves are shown here for *V*_*gate*_ higher than −1.0 V. The effect of electrical gating on the MoS_2_/GaAs solar cell is shown in Supplementary Information Fig. S4. The PCE values are 4.71%, 5.81% and 6.15% with the Voc values as 0.50 V, 0.55 V and 0.57 V corresponding to the *V*_*gate*_ values of 0 V, −0.5 V and −1.0 V, respectively. The *Φ*_*barrier*_ values correspondingly are 0.67 eV, 0.71 eV and 0.74 eV. The change of *Φ*_*barrier*_ is 0.07 eV, suggesting the Fermi level of MoS_2_ is shifted by 0.07 eV with the gate voltage of −1.0 V. For the MoS_2_/h-BN/GaAs device, Fermi level of MoS_2_ can be tuned by 0.10 eV as *Φ*_*barrier*_ is increased by 0.10 eV, 43% higher than that of the MoS_2_/GaAs device. In the 2D material/semiconductor heterostructure, change of the Fermi level of the 2D material can be suppressed by the charge transfer from GaAs substrate. In the MoS_2_/GaAs device, downshifting the Fermi level of MoS_2_ is inhibited by the electron transfer from GaAs to MoS_2_. By intersting h-BN to suppress the electron transfer, Fermi level tuning of MoS_2_ by the electrical gating can be more effective. From this point of view, interface h-BN can not only increase the initial *Φ*_*barrier*_ of the MoS_2_/h-BN/GaAs heterostructure, but also guarantees more effectively Fermi level tuning of MoS_2_.

## Discussion

MoS_2_/GaAs heterostructure based solar cell is investigated. Different from the traditional p-n junctions and metal/semiconductor Schottky junctions, the charge transfer between MoS_2_ and the GaAs substrate can greatly influence the position of Fermi level in the 2D material, which leads to a much lower barrier height than the ideal value originated from the Fermi level difference. The barrier height is a key factor for the electrical properties of electronic and optoelectronic devices. Thus, suppressing or preventing the charge transfer during the formation of the 2D material based heterojunctions is highly desired to achieve high performance devices. Herein, we demonstrated the performance of MoS_2_/GaAs based heterostructure solar cell is improved by inserting interlayer h-BN. The inserted h-BN layer can suppress the electron injection from n-type GaAs into MoS_2_ during the junction formation, while does not affect the hole separation and collection processes according to the electronic band structure of h-BN. Thus, PCE is increased from 4.82% to 5.42% after inserting the BN layer as higher barrier height and V_oc_ can be achieved. Furthermore, by employing chemical doping and electrical gating into the solar cell device, PCE of 9.03% is achieved, which is the highest among all the reported monolayer transition-metal dichalcogenide-based solar cells. This physical picture and technique could be extended into other 2D materials/semiconductor heterostructure based electronic and optoelectronic devices.

## Methods

Monolayer h-BN was grown on copper substrate with B_3_N_3_H_6_ as the precursor at 1000 ^o^C for 30 min^40^. Single layer MoS_2_ film was grown on Si/SiO_2_ substrate in a quartz tube with CVD method^41^. MoO_3_ powder and sulfur powder (99.9%, both bought from Aladdin) was used as the precursor. Growth temperature was set at 650 ^o^C. 60 nm Au was thermally evaporated on back surface of GaAs to form rear contact. GaAs substrate was cleaned by dipping the samples into 10%wt HCl solution for 5 min followed with DI water rinse. Surface passivation of GaAs was realized by remote NH_3_ plasma treatment for 5 min with 120 Watt 27.5 MHz RF generator. h-BN was transferred onto the GaAs substrate using PMMA as the sacrificing layer. After PMMA spun-on, MoS_2_ on Si/SiO_2_ substrate was immersed into deionized water to lift-off the PMMA-MoS_2_ films. After transferring, PMMA was removed by immersing the samples into acetone for 20 min.

MoS_2_ and h-BN were characterized by Raman spectroscopy (Renishaw inVia Reflex) with the excitation wavelength of 532 nm. The microstructure of MoS_2_ was examined by HRTEM (Tecnai F-20 operating at 200 KV). Atomic force Microscopy (AFM) characterization was performed using Veeco dimension 3100 system. The MoS_2_/h-BN/GaAs solar devices were tested by Agilent B1500A system with a solar simulator under AM1.5G condition. It is noteworthy that the illumination intensity was calibrated with a standard Si solar cell. Transient PL measurements were used to evaluate the charge recombination and separation behaviors at the interfaces of MoS_2_/h-BN/GaAs heterojunction. The excitation light source (PicoHarp 300 system) was a 450 nm pulsed laser with 1 MHz repetition rate and 50 ps pulse duration with power of 10 μW. The diameter of the excitation laser spot was 10 μm. The PL signal with wavelength shorter than 1100 nm was collected by a multimode optical fiber and recorded by a Horiba Jobin Yvon iHR550 spectrometer. All spectra were collected until the peak value reaching 5000 counts.

## Additional Information

**How to cite this article**: Lin, S. *et al.* Interface designed MoS_2_/GaAs heterostructure solar cell with sandwich stacked hexagonal boron nitride. *Sci. Rep.* **5**, 15103; doi: 10.1038/srep15103 (2015).

## Supplementary Material

Supplementary Information
