# Supplementary material for: Interface designed MoS2/GaAs heterostructure solar cell with sandwich stacked hexagonal boron nitride
Source: Sci Rep. 2015 Oct 13;5:15103. doi: 10.1038/srep15103 (PMC4602223; doi:10.1038/srep15103)
Supplement: Supplementary Information [file srep15103-s1.doc]

Supplementary Information:

Interface designed MoS_2_/GaAs heterostructure solar cell with sandwich stacked hexagonal boron nitride

Shisheng Lin,*†‡ Xiaoqiang Li, † Peng Wang, † Zhijuan Xu, †Shengjiao Zhang, † Huikai Zhong, † Zhiqian Wu, † Wenli Xu, † Hongsheng Chen*†‡

†Department of Information Science and Electronic Engineering, Zhejiang University, Hangzhou, 310027, China

‡ State Key Laboratory of Modern Optical Instrumentation, Zhejiang University, Hangzhou, 310027, China

Corresponding author: [shishenglin@zju.edu.cn](mailto:shishenglin@zju.edu.cn), [hansomchen@zju.edu.cn](mailto:hansomchen@zju.edu.cn)


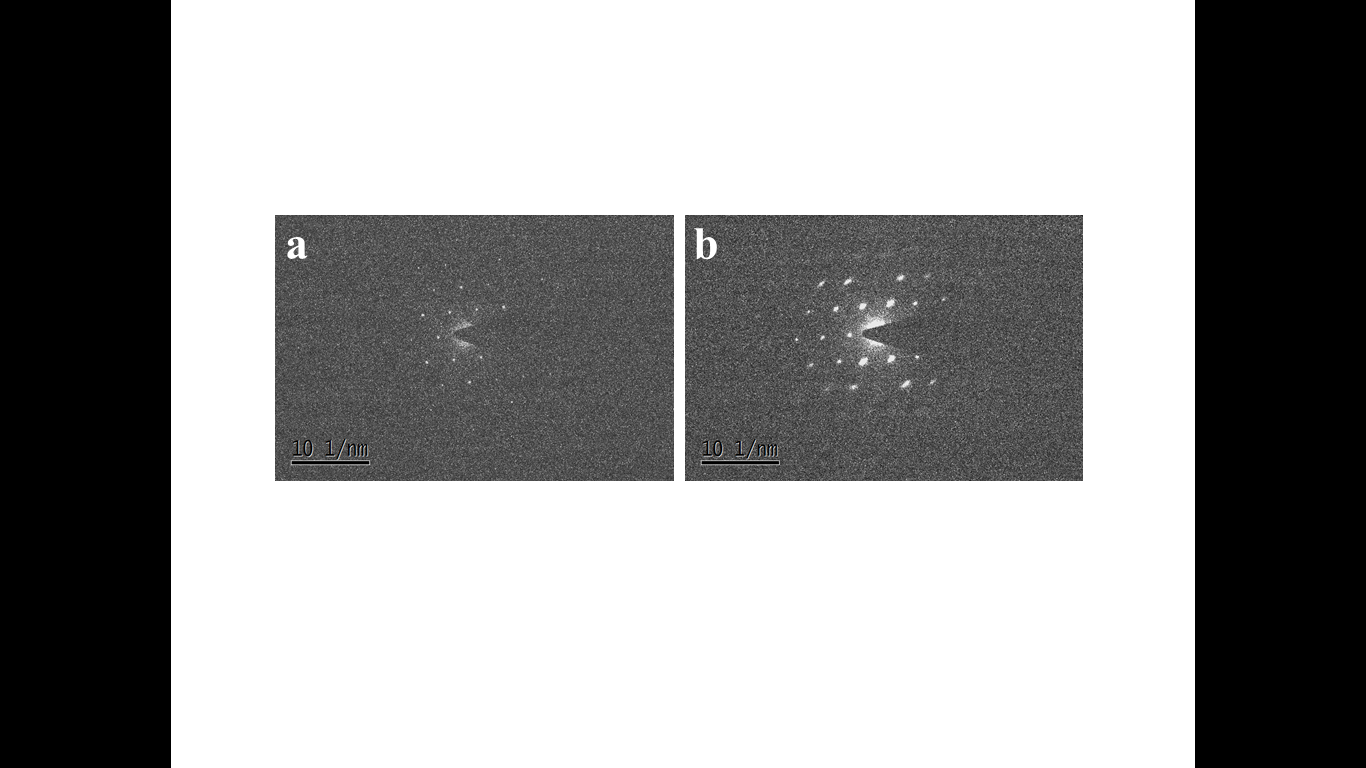


**Figure S1** ∣ Electron diffraction pattern of the MoS_2_ sheet with 0° tilt degree (a) and 30° tilt degree (b), which implies the monolayer nature of the 2D MoS_2_.


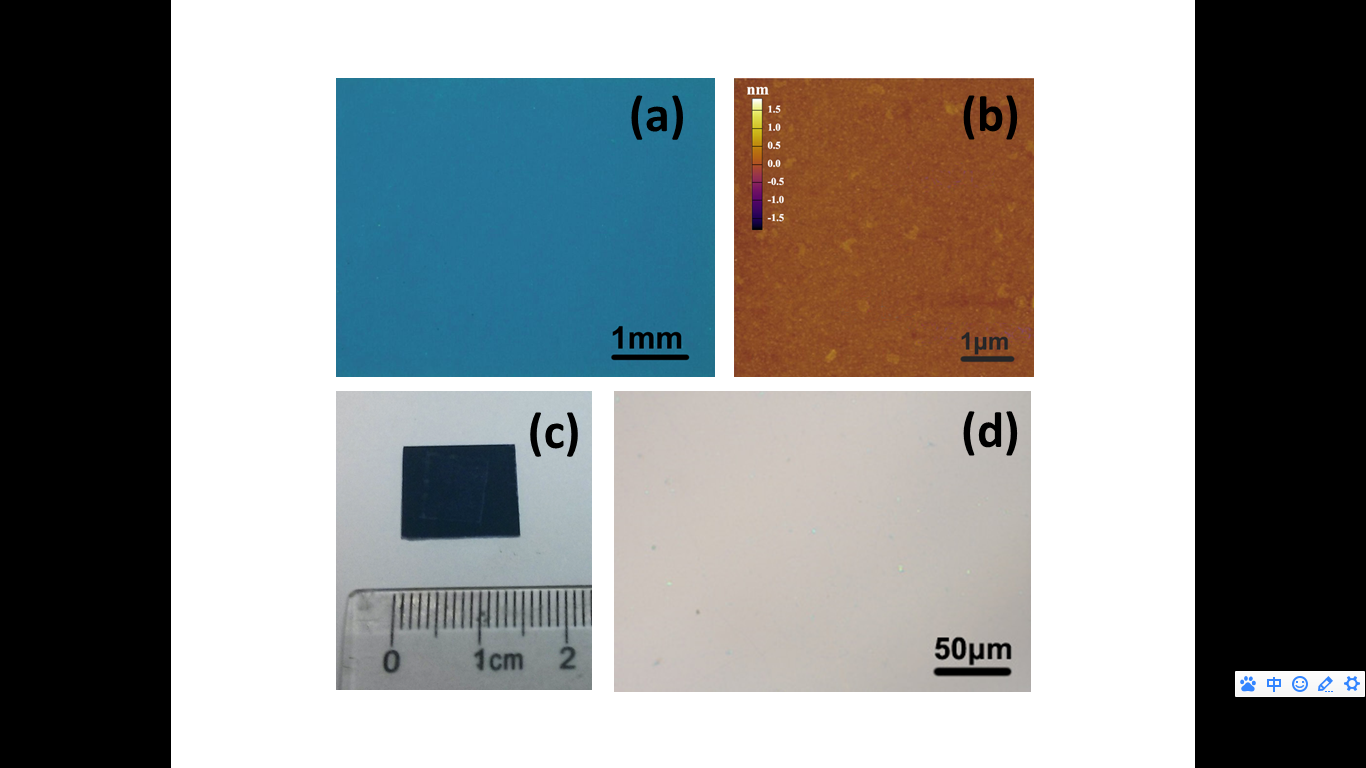


**Figure S2** ∣ (a) OM image of monolayer MoS_2_ grown on Si/SiO_2_ substrate. (b) AFM image of the monolayer MoS_2_. It is noteworthy that the measured thickness of the grown MoS_2_ is 0.68nm, in agreement with the monolayer MoS_2_. Digital photograph (c) and OM image (d) of the monolayer h-BN transferred on Si/SiO_2_ substrate.


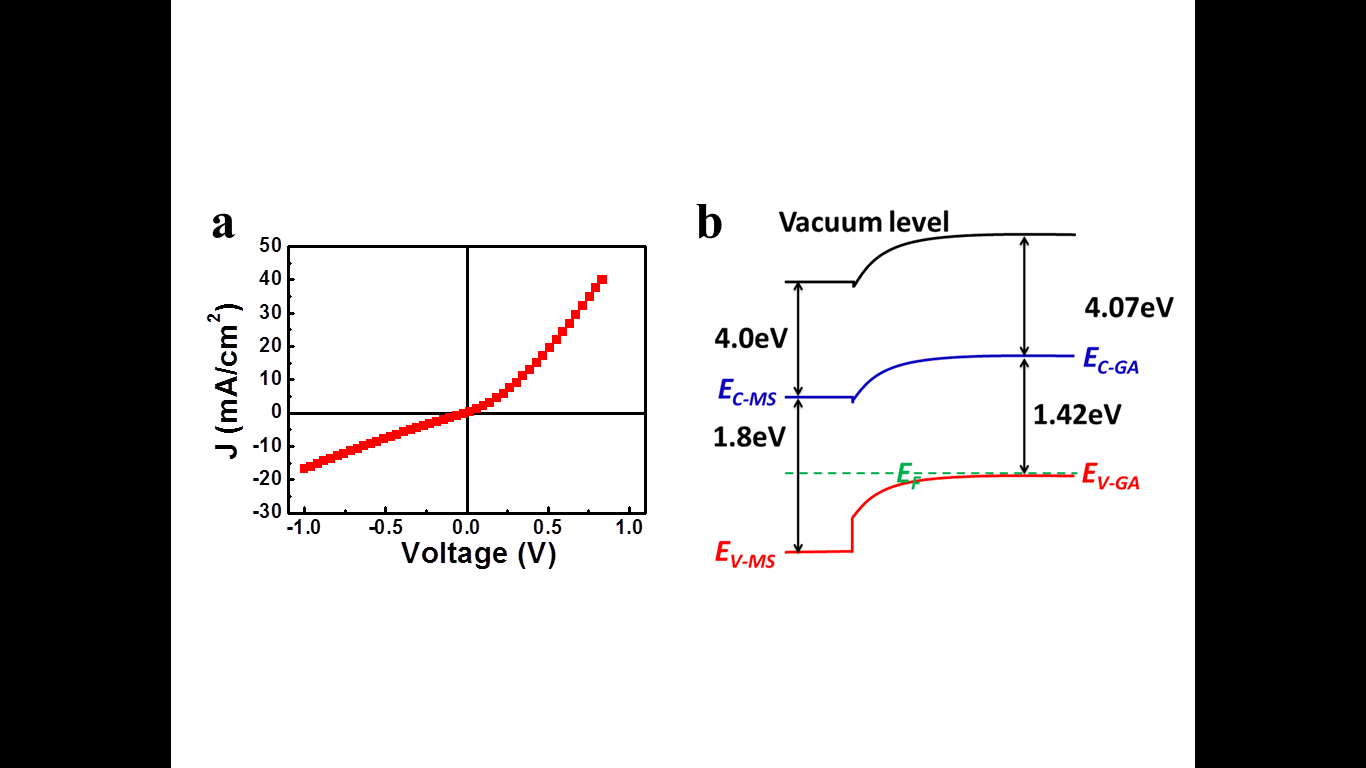


**Figure S3** ∣ (a) Typical dark *J*-*V* curve of the MoS_2_/p-GaAs heterojunction, which shows bad rectifying characteristics. (b) Schematic electronic band structure of the MoS_2_/p-GaAs heterojunction. Theoretically, MoS_2_ and p-type GaAs can form heterojuction with the Fermi level difference as 0.5eV when Fermi level of MoS_2_ locates at the middle of the band gap. While the measured *J*-*V* curve shows bad rectifying characteristics which may be attributed to that the interface defects states leads to low junction barrier height of the formed MoS_2_/p-GaAs heterojunction.


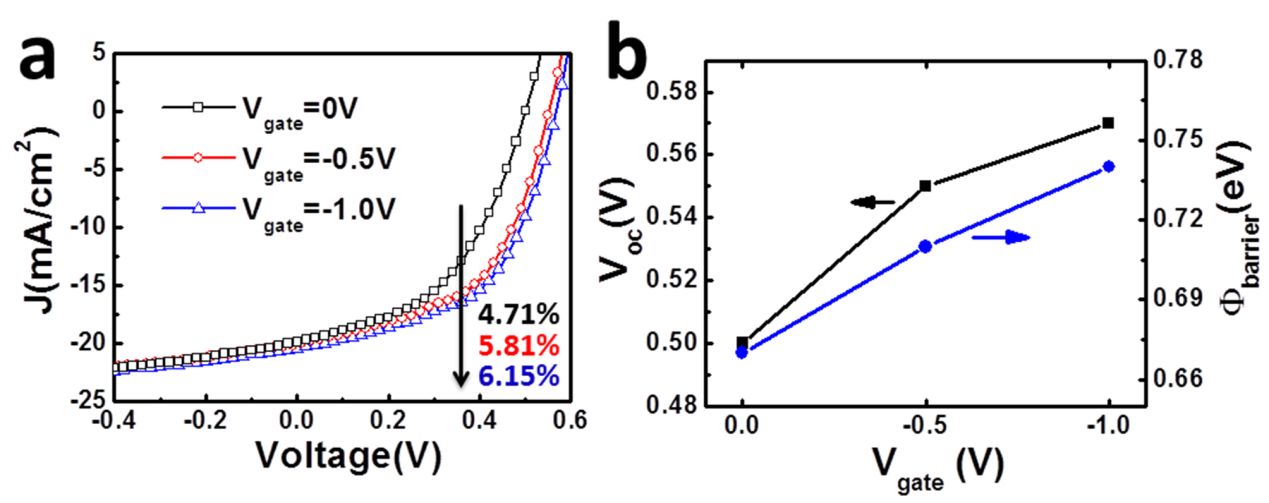


**Figure S4** ∣ (a) *J*-*V* curves of the field effect MoS_2_/GaAs heterojunctions under AM1.5G illumination and different *V_gate_*. (b) Values of *Φ_barrier_* and *V_oc_* correspond to different *V_gate_*.
